# Synaptojanin 1 Is Required for Endolysosomal Trafficking of Synaptic Proteins in Cone Photoreceptor Inner Segments

**DOI:** 10.1371/journal.pone.0084394

**Published:** 2014-01-02

**Authors:** Ashley A. George, Sara Hayden, Lars C. Holzhausen, Eva Y. Ma, Sachihiro C. Suzuki, Susan E. Brockerhoff

**Affiliations:** 1 Department of Biochemistry, University of Washington, Seattle, Washington, United States of America; 2 Department of Biological Structure, University of Washington, Seattle, Washington, United States of America; University Zürich, Switzerland

## Abstract

Highly polarized cells such as photoreceptors require precise and efficient strategies for establishing and maintaining the proper subcellular distribution of proteins. The signals and molecular machinery that regulate trafficking and sorting of synaptic proteins within cone inner segments is mostly unknown. In this study, we show that the polyphosphoinositide phosphatase Synaptojanin 1 (SynJ1) is critical for this process. We used transgenic markers for trafficking pathways, electron microscopy, and immunocytochemistry to characterize trafficking defects in cones of the zebrafish mutant, *nrc^a14^*, which is deficient in phosphoinositide phosphatase, SynJ1. The outer segments and connecting cilia of *nrc^a14^* cone photoreceptors are normal, but RibeyeB and VAMP2/synaptobrevin, which normally localize to the synapse, accumulate in the *nrc^a14^* inner segment. The structure of the Endoplasmic Reticulum in *nrc^a14^* mutant cones is normal. Golgi develop normally, but later become disordered. Large vesicular structures accumulate within *nrc^a14^* cone photoreceptor inner segments, particularly after prolonged incubation in darkness. Cone inner segments of *nrc^ a14^* mutants also have enlarged acidic vesicles, abnormal late endosomes, and a disruption in autophagy. This last pathway also appears exacerbated by darkness. Taken altogether, these findings show that SynJ1 is required in cones for normal endolysosomal trafficking of synaptic proteins.

## Introduction

Photoreceptors are highly specialized, polarized, light-sensitive cells in the retina. The apical end of the cell, the outer segment (OS), consists of stacked discs of membranes, which are the site of phototransduction. At the opposite end of the cell is the synaptic terminal, which is the site of neurotransmitter release. The inner segment (IS) of the cell is located between the OS and cell nucleus, and contains the cellular machinery required for production of energy and cellular components [1], [2]. Due to their highly polarized structure, photoreceptors must be able to properly sort and transport proteins made in the IS to opposite ends of the cell. The cell must also have mechanisms of removing damaged proteins that have reached their correct subcellular locations. In the case of OS proteins, damaged proteins are removed apically when discs are shed [3]. Because altered OS trafficking often results in photoreceptor degeneration, studies of protein trafficking in photoreceptors have focused mainly on trafficking of proteins from the Golgi in the IS to the OS [4], [5]. In contrast, little is known about the mechanisms used to sort and traffic proteins to the photoreceptor synapse, as well as the recycling and degradation of these proteins.

The zebrafish visual mutant *no optokinetic response c (nrc^a14^)* was identified in a mutagenesis screen [6]. The synaptic terminals of *nrc^a14^* cone photoreceptors have a flattened morphology, decreased number of synaptic vesicles, and unanchored synaptic ribbons [6]–[9]. The causative mutation was determined to be in the gene *synJ1*, resulting in the loss of Synaptojanin1 (SynJ1) protein [8]. SynJ1 is a polyphosphoinositide phosphatase with an established role in synaptic vesicle recycling. In conventional neurons, SynJ1 is concentrated at presynaptic terminals, where it is involved in the hydrolysis of phosphatidylinositol 4,5-bisphosphate (PI(4,5)P_2_) and the uncoating of clathrin-coated vesicles [10]. Interestingly, there is not an accumulation of clathrin coated vesicles in *nrc^a14^* cone photoreceptor terminals, suggesting a unique role for SynJ1 at ribbon synapses [6], [8]. SynJ1 is also present in the cell body and dendrites of neurons [11], [12]. While SynJ1 has been found to play a role in regulating endocytosis of AMPA receptors postsynaptically in dendrites [13], its function in the cell body of neurons has been less well studied. A recent study found that abnormal endosomal structures accumulate in the cell bodies of neurons from mice overexpressing SynJ1, suggesting that the functional role of SynJ1 is not exclusive to synaptic terminals [14].

We have previously shown that SynJ1 protein is present in the IS of cone photoreceptors in both adult and larval wild type (WT) zebrafish. This previous study also demonstrated that the synaptic protein VAMP2 localizes to the IS of *nrc^a14^*, but not WT, cone photoreceptors [9]. Together, these initial results suggested that SynJ1 also has important functions in the IS of zebrafish cone photoreceptors. In this study, we characterized the IS and trafficking defects associated with the loss of SynJ1. We report that the OSs and connecting cilia of *nrc^a14^* cone photoreceptors are morphologically normal. In contrast, select synaptic proteins and large vesicular structures accumulate in ISs. To dissect the cause of this accumulation, we analyzed markers for biosynthetic and degradative trafficking pathways. Whereas trafficking pathways for newly-synthesized proteins were not severely affected, there was an aberrant distribution and accumulation of acidic vesicles, late endosomes, and autophagosomes. Our findings show a segregation of apical and basal trafficking pathways in cones, and uncover the important role of SynJ1 in trafficking of synaptic proteins that depend on endolysosomal trafficking pathways. Together these results support the hypothesis that SynJ1 is required for proper membrane and protein trafficking at both the synapse and IS.

## Results

### Loss of SynJ1 does not Affect Cone Photoreceptor Connecting Cilia or Outer Segments

**Figure 1:**
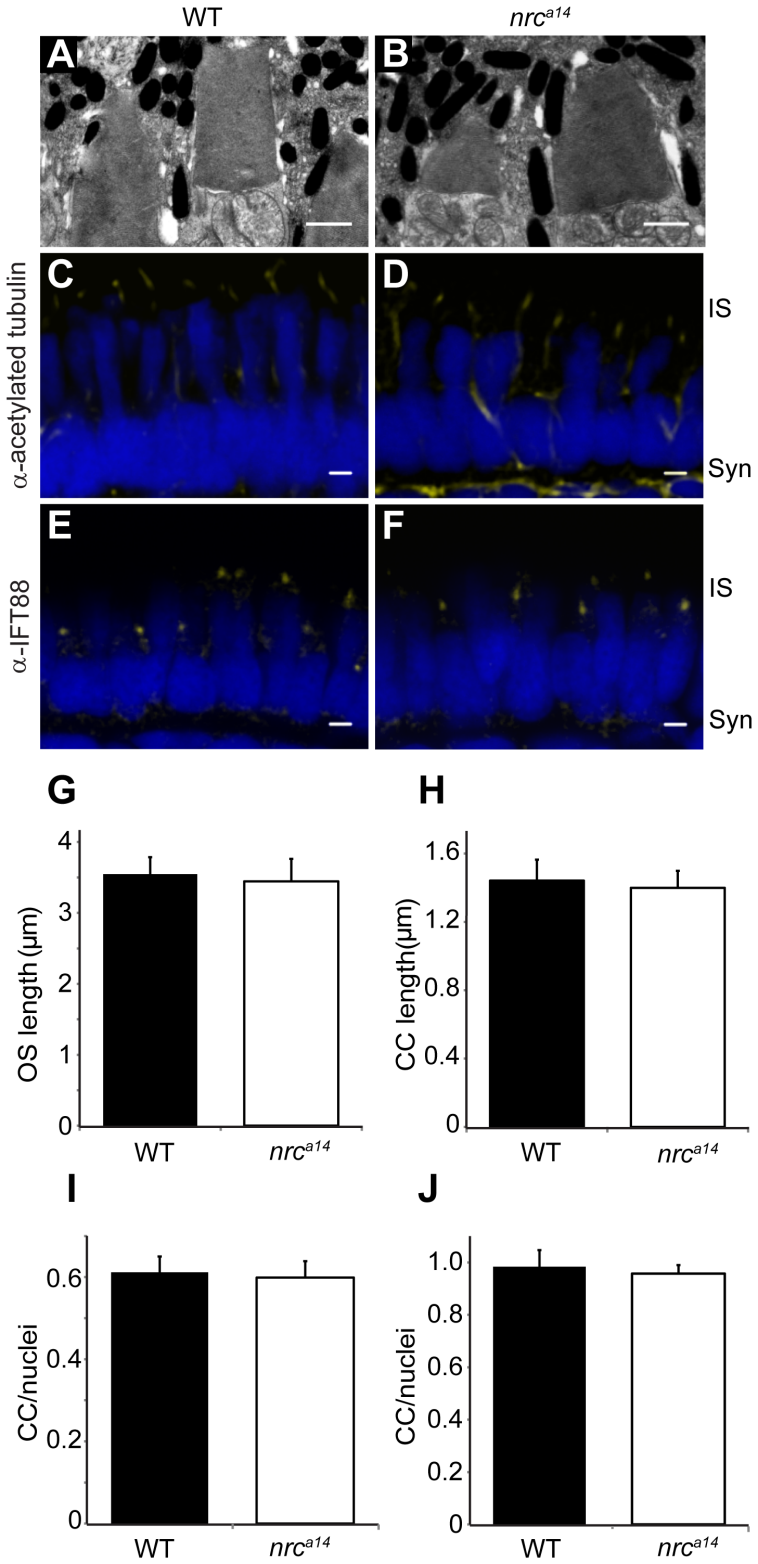
*nrc^a14^* cone photoreceptors have normal outer segments and connecting cilia. (A–B) TEM images of 5 dpf WT (A) and *nrc^a14^* (B) cone photoreceptor OSs. There was no difference in OS appearance or length (G) between *nrc^a14^* and WT cone photoreceptors at 5 dpf (p = 0.7). (C–F) Confocal images of 5 dpf zebrafish larval retinas immunostained using antibodies against the CC proteins acetylated tubulin (C, D) or IFT88 (E, F). The number (I) and length (H) of acetylated tubulin stained CC was not significantly different between WT and *nrc^a14^* photoreceptors (p = 0.9 and 0.4 respectively). There was no significant difference (p = 0.7) in the number of IFT88 stained cilia between WT and *nrc^a14^* (J). Antibody staining is shown in yellow; nuclei were stained with Sytox Green (D, E) or Hoechst (G, H) and are shown in blue. Syn = photoreceptor synapses, IS = inner segment. Scale bar = 1 µm in A and B and 2 µm in C–F. Graphs show mean values, error bars are STDEV for multiple larvae.

Defects in trafficking of proteins to the OS results in morphologically abnormal OSs and often leads to photoreceptor degeneration [15], [16]. To identify possible apical trafficking defects in *nrc^a14^* mutants, we performed transmission electron microscopy (TEM) on *nrc^a14^* mutant larvae and their WT siblings at 5 days postfertilization (5 dpf), and examined the morphology of the apical ends of their cone photoreceptors. At this age, WT larvae have robust visual responses that can be measured by optokinetic response (OKR) assays and electroretinogram recordings [17]–[19], and *nrc^a14^* behavioural and synaptic phenotypes are apparent [6]–[9]. TEM images demonstrated that the OSs of *nrc^a14^* cone photoreceptors appeared indistinguishable from their WT siblings. The OSs of both WT and *nrc^a14^* cone photoreceptors consist of characteristic, neatly-stacked membranes (Figure 1A, B). The OSs of *nrc^a14^* cone photoreceptors also showed no differences in length compared to WT cone OSs (Figure 1G; n = 100 cells from 4 larvae for both WT and *nrc^a14^*). These data suggest that protein transport to the OS proceeds normally in the absence of SynJ1.

As further verification of normal apical transport, we examined the structure of the connecting cilia (CC), a microtubule-based structure that connects the inner and outer segments. OS proteins are synthesized in the IS and transported through the CC [4]. We examined the CC using immunohistochemical staining against two components, acetylated tubulin [20] and IFT88 [21]. Acetylated tubulin is a structural component of the CC, while IFT88 is a component of the intraflagellar transport complex and is required for the formation and maintenance of OSs. Confocal images of either marker showed the presence of CC within both WT and *nrc^a14^* photoreceptors (Figure 1C–F). No difference was detected in the number of CC structures in WT and *nrc^a14^* cone photoreceptors with either acetylated tubulin or IFT88 (Figure 1I, J; n = 264–502 cells from 4–5 larvae). Because acetylated tubulin is found along the entire length of the CC, staining with this marker also allowed us to determine the length of CC in WT and *nrc^a14^* cone photoreceptors. We observed no difference in length of CC structures in WT and *nrc^a14^* cone photoreceptors (Figure 1E, F, H; n = 176–214 CC from 4–5 larvae). These results demonstrate that SynJ1 is not required for proper trafficking of proteins to the CC or the OSs of cone photoreceptors.

### Loss of SynJ1 Results in Mislocalized Synaptic Proteins in Cone Photoreceptors

**Figure 2:**
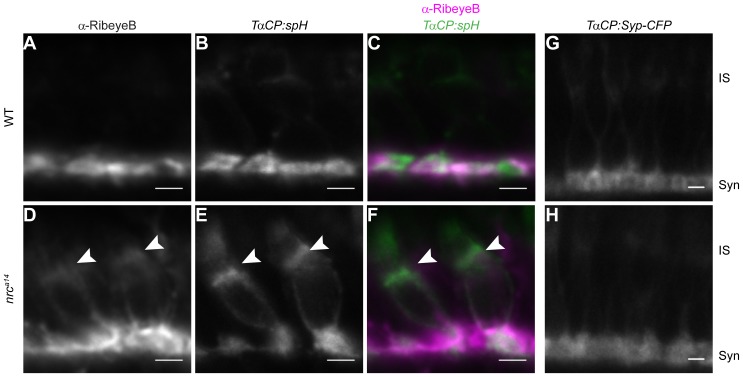
Some synaptic proteins are mislocalized in *nrc^a14^* cone photoreceptors. *Tg(TαCP:spH)* WT and *nrc^a14^* 5 dpf retinal slices were stained with an antibody against the ribbon synapse protein RibeyeB (A–F). In WT photoreceptors, RibeyeB was found only at synaptic terminals (A). In *nrc^a14^* cone photoreceptors, RibeyeB (D, arrowhead) and VAMP2 (E, arrowhead) were detected in both synaptic terminals and ISs. Mislocalized signals for RibeyeB (magenta) and VAMP2 (green) were coincident in the ISs of *nrc^a14^* cone photoreceptors (F, arrowhead). In contrast, the synaptic vesicle protein Synaptophysin (Syp-CFP) had a primarily synaptic distribution in both WT (G) and *nrc^a14^* (H) cone photoreceptors. Live confocal images were taken of 5 dpf *Tg(TαCP:Syp-CFP)* WT and *nrc^a14^* larvae. Syn = photoreceptor synapses, IS = inner segment. Scale bar = 2 µm.

Our previous studies focused on characterizing synaptic terminals of *nrc^a14^* cone photoreceptors [6]–[9]. We previously generated the transgenic fish strain *Tg(TαCP:spH)*, which expresses synaptopHluorin (spH) specifically in cone photoreceptors using the transducin alpha cone (TαCP) promoter [22]. SpH is a fusion of a pH-sensitive GFP and the synaptic vesicle SNARE protein VAMP2/synaptobrevin [23]. In WT larvae, spH localized to synaptic vesicles in cone synaptic terminals, whereas in *nrc^a14^* mutants, spH accumulated in the ISs and synaptic terminals ([9]; Figure 2B, E). To determine whether other synaptic proteins are mislocalized in *nrc^a14^* photoreceptors, we examined the distribution of two other proteins, RibeyeB and Synaptophysin. RibeyeB is an active zone protein and a component of the synaptic ribbon in zebrafish cone photoreceptors [24]. The distribution of endogenous RibeyeB was investigated by immunolabeling of retinal slices of 5 dpf *Tg(TαCP:spH)* larvae (Figure 2A, C, D, F). In WT retinas, RibeyeB was localized to photoreceptor synapses, consistent with previous reports ([24]; Figure 2A). In contrast, RibeyeB staining was visible at both the synapses as well as the ISs of *nrc^a14^* cone photoreceptors (Figure 2D, F). Further, the mislocalized RibeyeB detected in *nrc^a14^* ISs was coincident with the mislocalized spH (Figure 2F). This mislocalization of endogenous RibeyeB was not due to overexpression of spH, as identical RibeyeB staining patterns were observed in both non-transgenic WT and *nrc^a14^* cone photoreceptors (Figure S1).

Synaptophysin is a synaptic vesicle glycoprotein commonly used as a presynaptic marker [12], [25]. To examine the distribution of Synaptophysin, we generated the transgenic fish line *Tg(TαCP:Syp-CFP)*, which expresses CFP- tagged zebrafish Synaptophysin in cone photoreceptors. At 5 dpf, Synaptophysin-CFP was observed primarily at the synapse, but also in small amounts in the ISs of both WT and *nrc^a14^* cone photoreceptors. There was no apparent difference in its distribution between *nrc^a14^* and WT cone photoreceptors (Figure 2G, H). The detection of Synaptophysin at both the synapse and the IS of WT photoreceptors is consistent with previous findings [26]. This result indicates that not all synaptic proteins are mislocalized in *nrc^a14^* cone photoreceptors, and suggests that there may be multiple pathways for trafficking synaptic proteins in cone photoreceptors, only some of which require SynJ1.

### Large Vesicular Structures Accumulate in *nrc^a14^* Inner Segments

**Figure 3:**
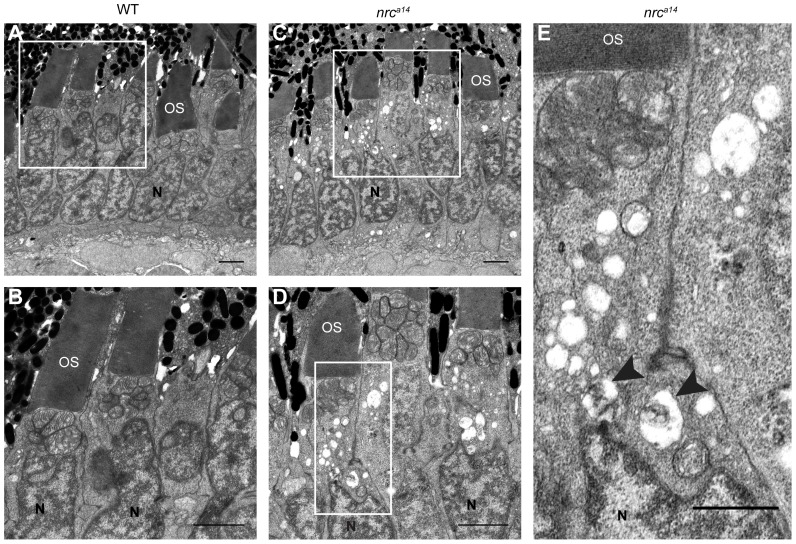
Large vesicular structures accumulate in *nrc^a14^* photoreceptor inner segments. TEM images of cone photoreceptors in WT (A, B) and *nrc^a14^* (C–E) 5 dpf retinas. Large vesicular structures were visible in approximately 80% of *nrc^a14^* cone photoreceptor ISs. Some vesicular structures contained electron dense material (arrow heads, E). Boxes in A and C show areas enlarged in B and D respectively. Box in D shows area enlarged in E. OS = outer segment, N = nuclei. Scale bar = 2 µm in A–D, 1 µm in E.

The presence of SynJ1 protein in the IS of WT cones and the presence of ectopic synaptic proteins in the IS of *nrc^a14^* cones suggest that SynJ1 plays a functional role at the IS of cone photoreceptors. Therefore, we analyzed the morphology of the *nrc^a14^* cone IS in 5 dpf larvae in more detail using TEM. We made the surprising finding that the IS of *nrc^a14^* cone photoreceptors contained large vesicular structures that were not often observed in WT cone photoreceptors (Figure 3). These structures were present in approximately 80% of *nrc^a14^* cone photoreceptors and were variable in size, ranging from ∼125 to 900 nm in diameter (n = 547 cells from 4 *nrc^a14^* larvae). While many of these structures were devoid of electron density, a few contained electron-dense material (Figure 3E, arrowheads).

Photoreceptors are depolarized in the dark and release glutamate from their synaptic terminals. Both vesicle trafficking to the synapse and recycling of synaptic vesicles would be expected to increase in the dark. Although *nrc^a14^* photoreceptors show altered synaptic activity, they respond to light [7]. Therefore, we hypothesized that *nrc^a14^* photoreceptors should similarly modulate trafficking of synaptic proteins between the IS and the synaptic terminal in response to changing light conditions. Furthermore, previous work on zebrafish hair cells lacking SynJ1 has shown that the abnormal, blebbed synaptic morphology of these sensory neurons is dependent upon synaptic activity [27]. To investigate whether the IS phenotype of cone photoreceptors lacking SynJ1 was similarly dependent on synaptic activity, we incubated 4 dpf WT and *nrc^a14^* larvae in the dark for 24 hours prior to fixation for TEM. As a control, light-adapted WT and *nrc^a14^* sibling larvae were kept on a 14/10 hr light/dark cycle for the same 24 hr period.

**Figure 4:**
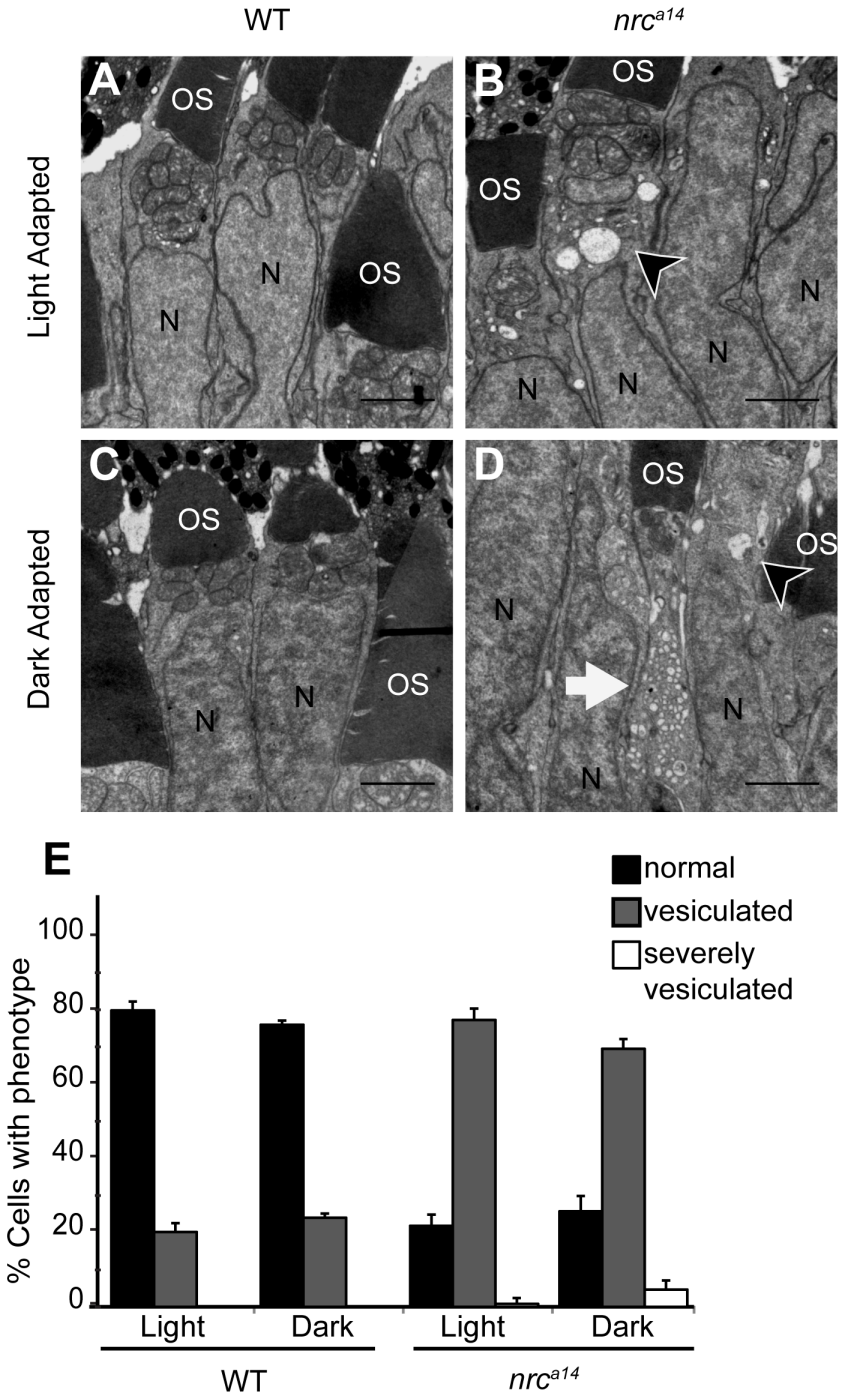
Figure 4. Dark adaptation increases the number of vesicular structures in *nrc^a14^* photoreceptor inner segments. TEM images of cone photoreceptors in light (A, B) or dark-adapted (C, D) WT and *nrc^a14^* retinas. At 4 dpf, larvae were phenotyped by OKR and placed at 28°C either on a normal light/dark cycle, or in complete darkness. 24 hours later, at 5 dpf, larvae were fixed for TEM. Dark adaptation exaggerated the vesicular structure phenotype in *nrc^a14^* cone photoreceptor inner segments (B vs. D). Cells were scored as “normal”, “vesiculated” or “severely vesiculated” and the quantification is shown in E. Cells with at least 3 vesicular structures >125 nm were scored as “vesiculated” and examples are shown by arrow heads in B and D, cells that contained at least 20 vesicular structures were scored as “severely vesiculated” and an example is shown by an arrow in D. OS = outer segment, N = nuclei. Scale bar = 2 µm in A–D. Graph shows mean, error bars are STDEV for three independent light/dark experiments. In total at least 400–500 cells from 5 larvae were counted per light condition and genotype.

In light-adapted (LA) WT retinas, the majority (80+/−2%) of cone photoreceptors contain “normal” ISs lacking large vesicular structures (Figure 4A and E; n = 504 cells from 5 larvae in 3 independent trials). In contrast, the majority (77+/−3%) of LA *nrc^a14^* photoreceptors were “vesiculated” and contained at least 3 large (>125 nm) vesicular structures (Figure 4B, arrowhead; n = 406 cells from 5 larvae in 3 independent trials). We found that 24 hours of darkness caused a slight, but not significant, increase in the number of WT cells with at least 3 vesicular structures in the IS (Figure 4E, ∼20% to ∼24%; n = 508 cells from 5 larvae in 3 independent trials; p = 0.2). In contrast, the effect of dark incubation on *nrc^a14^* ISs produced a dramatic phenotype change (Figure 4D, white arrow). While the total percentage of *nrc^a14^* cones containing vesicular structures was not affected by dark adaptation, 6+/−3% of cells with aberrant vesicular structures in the IS showed large increases in vesicle number with a concomitant decrease in vesicle size (Figure 4E; n = 411 cells from 5 larvae in 3 independent trials). These “severely vesiculated” cells had over 20 vesicles in the IS per cell and was never observed in WT LA or dark-adapted (DA) larval retinas and was rarely seen in LA *nrc^a14^* ISs (p = 0.077 for LA vs. DA *nrc^a14^*). These results suggest that SynJ1 is involved in trafficking pathways that are modulated during dark adaptation in cone photoreceptors.

### *nrc^a14^* Cone Photoreceptor Inner Segments have Normal Endoplasmic Reticulum but Acquire a Disordered Golgi Apparatus

Our experiments demonstrated that the trafficking pathway(s) affected in *nrc^a14^* mutant cone photoreceptors involve the trafficking of synaptic proteins and can be modulated by changes in photoreceptor activity in the dark. The accumulation of synaptic proteins and vesicles in the IS could be due to either defects in trafficking of newly-synthesized proteins to the synapse, or in the recycling and/or degradation of these proteins. To dissect the trafficking pathways affected in the *nrc^a14^* cone photoreceptors, we created constructs to express fluorescent markers targeted to various subcellular organelles. We first investigated the Endoplasmic Reticulum (ER) and the Golgi apparatus, which are both involved in the trafficking of newly-synthesized proteins. In addition, we examined the morphology of the Golgi apparatus at various developmental time points. We reasoned that a primary defect in trafficking newly synthesized proteins would result in Golgi disruption during photoreceptor cell differentiation, but prior to onset the of photoreceptor activity. However, if the primary defect is in pathways involved in protein recycling or degradation, we would see no disruption of the Golgi, or that Golgi disruption would occur as a secondary defect after the onset of photoreceptor synaptic activity.

To visualize the ER in cone photoreceptors, we used the ER-targeting sequence from the protein calreticulin [28] to generate a transgenic fish line expressing ER-targeted GFP specifically in cone photoreceptors (*Tg(TαCP:ER-GFP*)*)*. To visualize the morphology of the Golgi apparatus in cone photoreceptors, we generated the transgenic fish line *Tg(crx:Man2a-GFP)* which expresses GFP targeted to the medial Golgi. The *crx* promoter drives expression of the Golgi marker GFP in cones, rods, and bipolar cells at earlier time points in photoreceptor development than the *TαCP* promoter [29]. We confirmed that the marker Man2a-GFP resides in the Golgi by treating 3 dpf *Tg(crx:Man2a-GFP)* zebrafish larvae with Brefeldin A (BFA). BFA disrupts ER-to-Golgi trafficking and Golgi morphology, resulting in the redistribution of Golgi resident proteins to the ER [30]. After BFA incubation, the Man2a-GFP signal dispersed to both the ER and fragmented structures distributed throughout the cell (Figure S2). This observation indicates that the Man2a-GFP fusion protein mimics the behavior of endogenous medial Golgi proteins.

**Figure 5:**
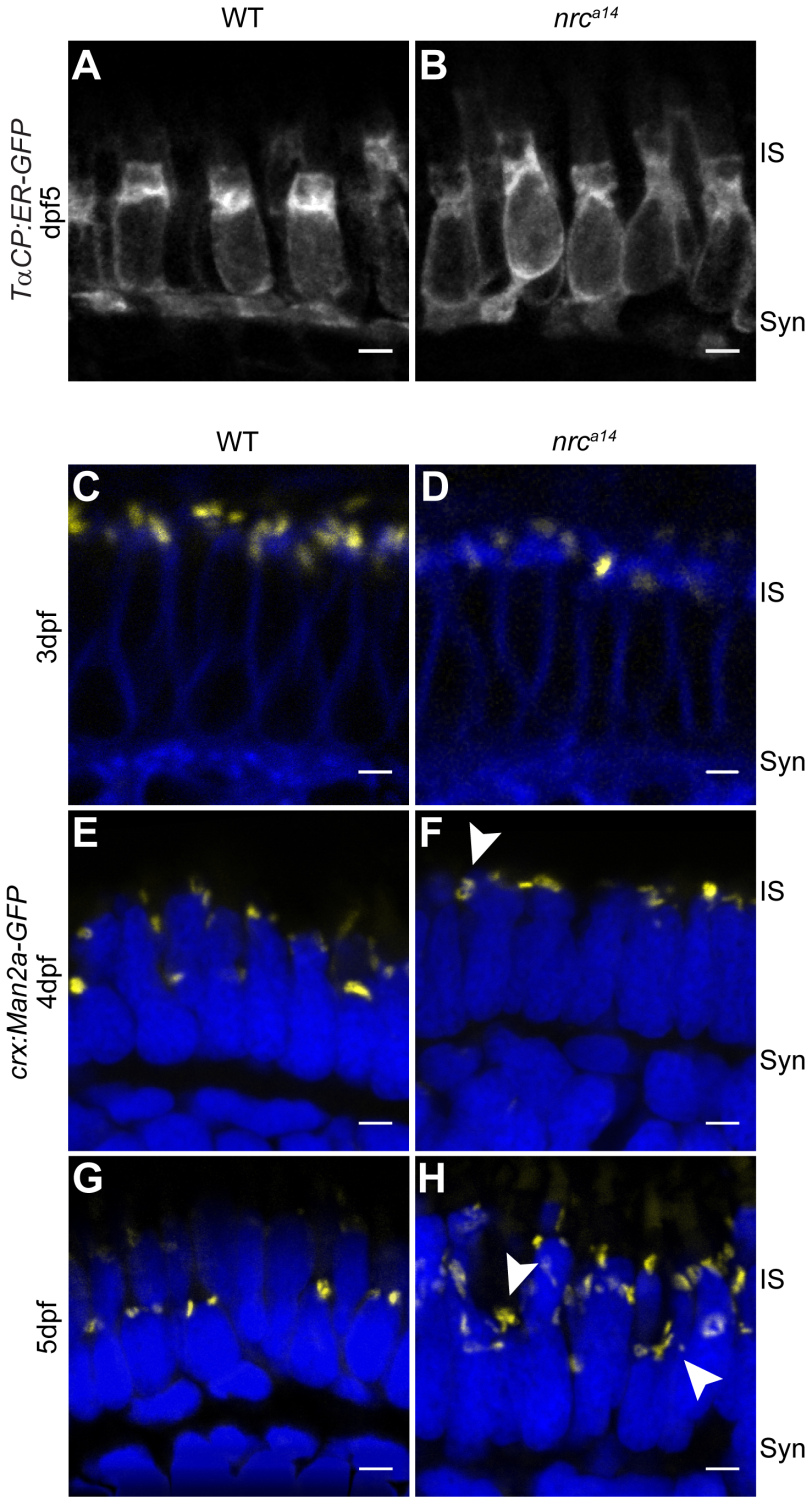
SynJ1 is required for Golgi maintenance but not development. The transgenic fish line *Tg(TαCP:ER-GFP)* was used to mark the ER. Retinal slices from 5 dpf WT (A) and *nrc^a14^* (B) larvae showed that the overall ER morphology was unaltered in the absence of SynJ1. Confocal images of *Tg(crx:Man2a-GFP)* WT (C, E, G) or *nrc^a14^* (D, F, H) larvae retina at 3–5 dpf showed that the Golgi is normal during photoreceptor development, but develops abnormalities disordered after cones become functional. No apparent abnormalities were seen in Golgi morphology of *nrc^a14^* compared to WT photoreceptors at 3 dpf (compare C and D). At 4 dpf, some mild morphology changes appeared in *nrc^a14^* Golgi (F, arrowhead). At 5 dpf, fragmented Golgi were visible in *nrc^a14^* photoreceptors (H, arrowheads). Man2a-GFP signal is shown in yellow, membranes of 3 dpf larvae were stained with BODIPY-TR and are shown in blue, and nuclei were stained with Hoechst and are shown in blue for 4 dpf and 5 dpf images. Syn = photoreceptor synapses, IS = inner segment. Scale bar = 2 µm in all images.

Confocal microscopy of retinal slices from 5 dpf larvae demonstrated no gross differences in ER morphology between WT and *nrc^a14^* cone photoreceptors (Figure 5A, B), suggesting that the membranes observed in the TEM images (Figures 3 and 4) were not dilated or disordered ER. Next, we examined WT and *nrc^a14^* retinas from *Tg(crx:Man2a-GFP)* larvae using confocal microscopy to evaluate Golgi morphology between 3 dpf and 5 dpf (Figure 5C–H). WT photoreceptors contained rounded or elongated Golgi structures which were located in an ordered manner in the IS on top of the cell nuclei at every time point investigated (Figure 5C, E, G). At 3 dpf, Golgi structures in *nrc^a14^* photoreceptors also appeared as rounded structures located at the apical side of the nucleus (Figure 5D). At 4 dpf a few disordered Golgi became visible in *nrc^a14^* photoreceptors (Figure 5F, arrowhead). By 5 dpf the Golgi appear disordered and fragmented in *nrc^a14^* cone photoreceptors (Figure 5H, arrowheads). We also examined the fish line *Tg(TαCP:Man2a-GFP)* (data not shown) which expresses the Golgi marker in only cone photoreceptors and observed similar disordered Golgi structures. Therefore we can conclude that the disordered structures we see are present within cone photoreceptors. These data suggest that SynJ1 is not required for the development of the Golgi apparatus in *nrc^a14^* cone photoreceptors, but is required for the maintenance of Golgi structures after 3 dpf.

We had initially hypothesized that the vesicular structures observed in TEM images of *nrc^a14^* photoreceptors could be disordered Golgi membranes. However, our confocal data (Figure 5) suggested that the vesicular structures observed in the TEM images were not derived solely from medial Golgi membranes. The vesicular structures in the TEM images occupied a significant portion of the cone photoreceptor IS. In contrast, the Man2a-GFP-positive structures observed with confocal microscopy did not appear to occupy the same volume of the IS as the vesicular structures (compare Figures 3 & 4 with Figure 5), suggesting that these membranes may be derived from other disrupted trafficking pathways.

### *nrc^a14^* Cone Photoreceptors have Abnormal Late Endosomes and an Increase in Autophagosomes

Photoreceptors depend on high membrane and protein turnover for their survival and thus defects in the late endosomal degradative pathway would likely cause severe membrane trafficking abnormalities. Furthermore, this pathway is tightly regulated by phosphoinositides [31]. To analyze the endolysosomal system, we used LysoTracker Red and followed two major molecules in the degradative pathway, Rab7 and LC3. LysoTracker Red accumulates in acidic organelles and has been used to label lysosomes in live zebrafish larvae [32]. Rab7 is a GTPase involved in late endosomal trafficking [33], lysosome biogenesis [34], maturation of late autophagic vacuoles [35] and is commonly used as a marker of the late endolysosomal pathway [36]. LC3 is a protein involved in the formation of autophagosome membranes. Changes in the presence of LC3-positive puncta are indicative of alterations in autophagy [37]. To investigate whether late endosomal trafficking pathways are disrupted in *nrc^a14^*cone photoreceptors we generated transgenic fish lines that target GFP to late endosomes (*Tg(TαCP:GFP-Rab7)*) or autophagosomes (*Tg(TαCP:GFP-LC3*)).

**Figure 6:**
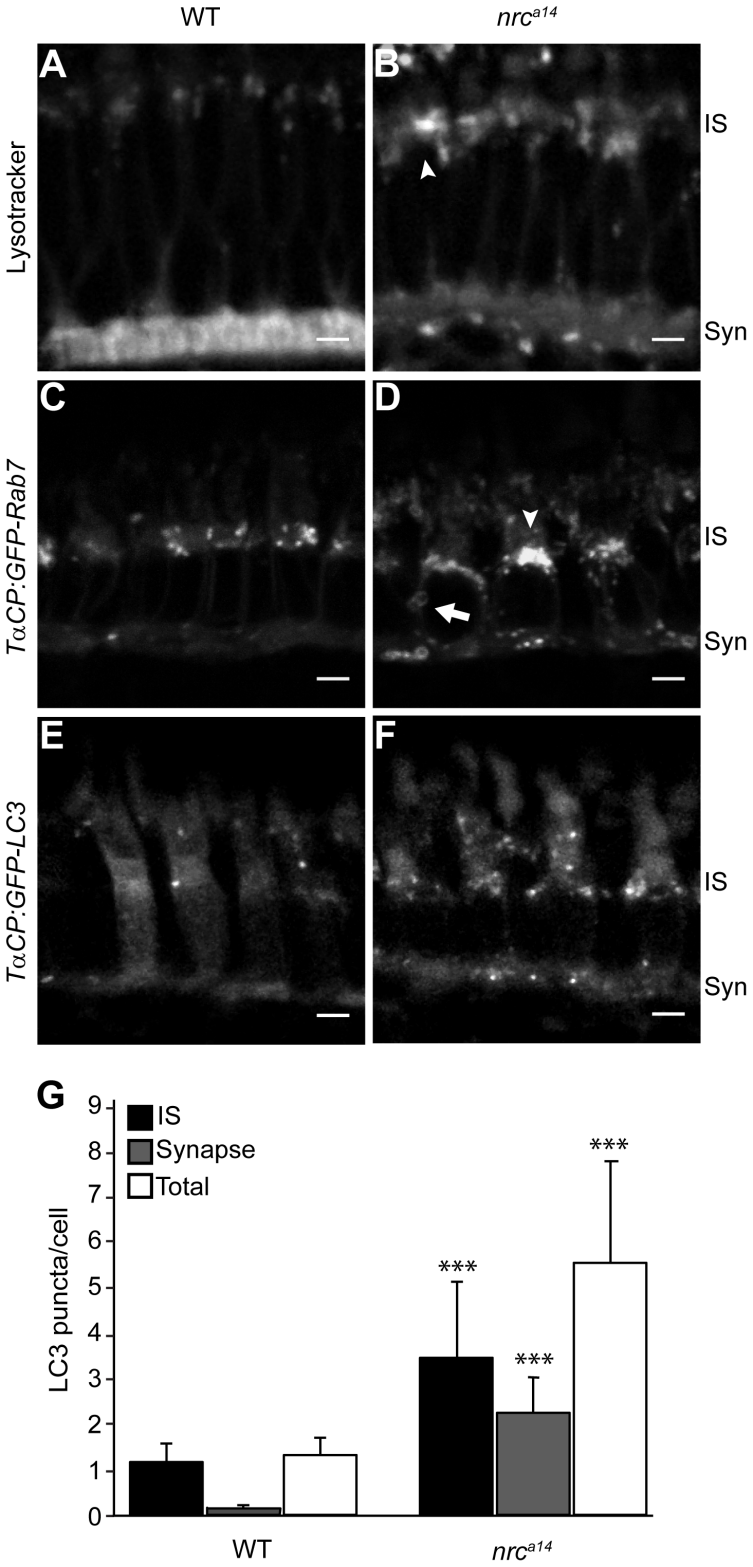
Loss of SynJ1 disrupts endolysosomal structures. Confocal images of larvae incubated with Lysotracker Red and the transgenic fish lines *Tg(TαCP:GFP-Rab7)* and *Tg(TαCP:GFP-LC3)* mark the endolysosomal system. 5 dpf larvae were incubated in Lysotracker and imaged live. In WT retinas (A), Lysotracker Red accumulated primarily in the synapse and in small punctate structures in the IS of cone photoreceptors. In *nrc^a14^* cone photoreceptors (B), Lysotracker Red accumulated primarily in larger, abnormal structures in the IS. Retinal slices from WT (C) and *nrc^a14^* (D) larvae show that abnormal Rab7-positive structures including large perinuclear structures (arrowheads), and doughnut shaped structures (arrows) accumulated in the IS and synapse in the absence of SynJ1. The lack of SynJ1 also caused an increase in the number of LC3-GFP-positive structures, indicating a disruption in autophagy in *nrc^a14^* cones (F). The number of LC3 puncta is increased in both the IS and synapse of *nrc^a14^* cones (G). Syn = photoreceptor synapses, IS = inner segment. Scale bar = 2 µm in all images. Graph shows average LC3 puncta in the IS, synapse or entire cell (Total) per cell, error bars are STDEV for 11 WT larvae and 12 *nrc^a14^* larvae. The number of LC3 puncta per cell for each subcellular compartment or the entire cell was significantly different between WT and *nrc^a14^* larvae (p-value<0.001, donated by ***).

Lysotracker Red staining in WT photoreceptors was observed primarily in synaptic vesicles at the synapse, but also in small punctate structures in the IS (Figure 6A). In contrast, the majority of the Lysotracker Red accumulated in larger, globular structures in the IS of *nrc^a14^* photoreceptors (Figure 6B, arrowhead) with very little staining at the synapse. The lack of staining at *nrc^a14^* photoreceptor synapses is consistent with our previous findings of synaptic vesicle defects in *nrc^a14^* cone photoreceptors [6].

In WT cone photoreceptors, Rab7 positive structures appeared punctate and localized primarily to the IS (Figure 6C). In contrast, we found that *nrc^a14^* cone photoreceptors contained abnormal Rab7-positive structures (Figure 6D). While some of the Rab7 structures in *nrc^a14^* cone photoreceptors appeared punctate and similar in appearance to those in WT cells, the majority of the cells had large perinuclear and/or doughnut shaped structures (Figure 6D, arrowheads and arrow respectively) that were not observed in WT cone photoreceptors. There was also an overall increase in the number of Rab7 structures in *nrc^a14^* cone photoreceptors compared to WT (Figure 6C, D).

Upon induction of autophagosome formation, the distribution of LC3 changes from diffusely cytosolic to punctate as it becomes concentrated in autophagosome membranes. The GFP-LC3 signal in cone photoreceptors from WT larvae was primarily cytosolic. Few GFP-LC3 puncta were observed in WT cells, with the majority of the GFP-LC3 puncta located in the IS (Figure 6E, G; average of 1.3+/−0.4 puncta/cell; median of 1 puncta/cell; n = 823 cells from 11 larvae). In contrast, *nrc^a14^* cone photoreceptors contained many more GFP-LC3 puncta. In addition, the GFP-LC3 puncta in *nrc^a14^* cone photoreceptors were more broadly distributed throughout the IS and at the synapse (Figure 6F, G; average of 5.5+/−2.2 puncta/cell; median of 4 puncta/cell; n = 1055 cells form 12 larvae; p = 0.000001). An increase in GFP-LC3 puncta could be indicative of an increase of autophagosome formation, or a decrease in autophagosome clearance.

Together, the abnormalities detected in *nrc^a14^* cones using all three markers of the endolysosomal/autophagy pathway indicate that the loss of SynJ1 significantly disrupts this important trafficking pathway in cone photoreceptors.

**Figure 7:**
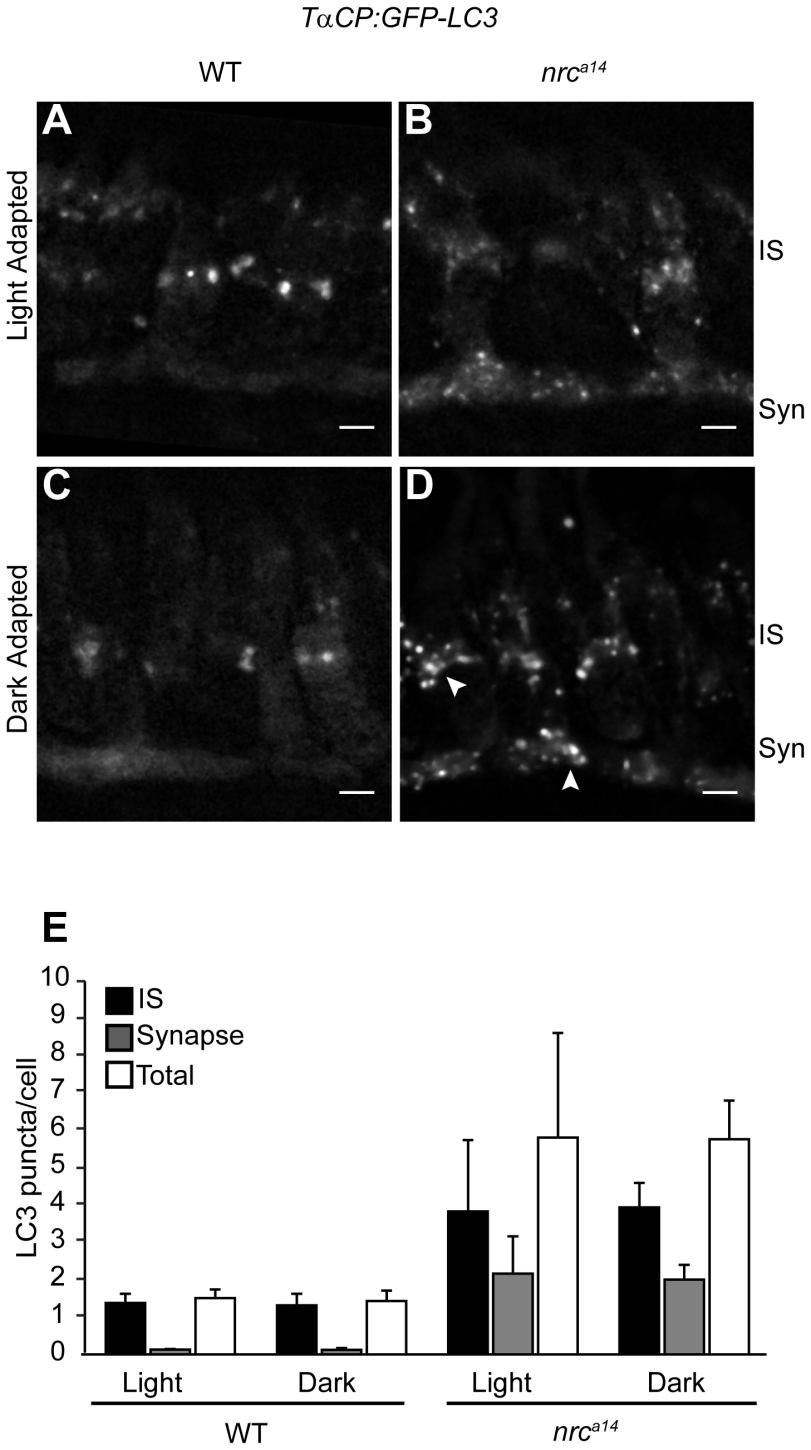
Dark adaptation affects autophagosomes in *nrc^a14^* cone photoreceptors. Confocal images of cone photoreceptors in light (A, B) or dark-adapted (C, D) WT and *nrc^a14^ Tg(TαCP:GFP-LC3)* retinas. At 4 dpf, larvae were phenotyped by OKR and placed at 28°C either on a normal light/dark cycle, or in complete darkness. 24 hours later, at 5 dpf, larvae were fixed and retinal slices were generated. After dark incubation, the GFP-LC3 positive puncta in *nrc^a14^* cone photoreceptors appeared enlarged (D, arrowhead). Dark incubation did not significantly affect the number of GFP-LC3 puncta in WT or *nrc^a14^* cone photoreceptors (E). Syn = photoreceptor synapses, IS = inner segment. Scale bar = 2 µm in all images. Graph shows average LC3 puncta in the IS, synapse or entire cell (Total) per cell, error bars are STDEV, from two independent light/dark experiments. For each experiment, 3–5 larvae were used per condition, and 60–150 cells were analyzed per larvae.

In order to correlate the vesicular structures observed in our TEM images with the autophagic trafficking pathway, we repeated our light/dark experiments using our *Tg(TαCP:GFP-LC3*) fish line. An accumulation of GFP-LC3-positive puncta after dark incubation of *nrc^a14^* larvae similar to the accumulation of vesicles seen in the “severely vesiculated” photoreceptors in Figure 4 would provide strong evidence that the vesicular structures observed in TEM images of *nrc^a14^* photoreceptors are in the autophagic trafficking pathway. WT and *nrc^a14^* sibling larvae were incubated in complete darkness, or left on a normal light/dark cycle as described above for 24 hours prior to fixation and quantification of GFP-LC3 puncta. We found that dark incubation did not significantly increase the average number of GFP-LC3 puncta in either WT or *nrc^a14^* cone photoreceptors (Figure 7E, n = 2 independent light/dark trials, 3–5 larvae per condition, 60–100 cells per larvae). However, we observed that dark incubation changed the morphology of GFP-LC3 puncta in *nrc^a14^* cone photoreceptors (Figure 7C, D). While light-adapted *nrc^a14^* larvae contained numerous small GFP-LC3 puncta, dark adapted *nrc^a14^* cone photoreceptors contained larger, more intense puncta and less diffuse GFP-LC3 in the cytoplasm (Figure 7D, arrowhead). At the level of confocal resolution, it is unclear whether these larger puncta are due to an increase in size of autophagosomes or large aggregates of puncta that cannot be distinguished from one another. These light/dark experimental results together indicate that increasing synaptic activity with dark adaptation alters both the appearance of vesicular structures in the TEM images (Figure 4) as well as the morphology of GFP-LC3 puncta in *nrc^a14^* cone photoreceptors (Figure 7),

## Discussion

Proteins synthesized in the IS of cone photoreceptors are sorted and transported apically toward the OS or basally toward the synapse. Dysfunctional folding, sorting, or trafficking of OS and ciliary proteins are frequently the cause of photoreceptor degeneration (for example [38]). In addition, OS membranes and proteins are continually shed and must be replaced, resulting in this compartment having a very high demand for protein trafficking. As a consequence, the mechanisms involved in apical transport have been the focus of much more attention than those involved in synaptic protein transport in photoreceptors.

In this study, we identified a novel role for SynJ1 in the IS of cone photoreceptors. We characterized IS trafficking defects in the *nrc^a14^* zebrafish mutant, which lacks SynJ1. Loss of SynJ1 caused an apparent defect in trafficking of the synaptic proteins VAMP2 and RibeyeB, but no defect in apical trafficking of OS or CC proteins. We extended this analysis and determined that late endolysosomal trafficking pathways are disrupted in *nrc^a14^* mutant cones. These data suggest that SynJ1 does not play a role in general protein trafficking, but rather a specific role in sorting or transporting synaptic proteins that rely on the endolysosomal and autophagic systems.

SynJ1 is a polyphosphoinositide phosphatase with an established role in synaptic vesicle recycling in conventional neurons. The SynJ1 protein is highly conserved, and consists of two phosphatase domains, a 5′Ptase and a Sac1 domain, and a proline rich C-terminal region involved in protein-protein interactions [11]. Mouse *synJ1* knockouts show significantly elevated levels of PI(4,5)P_2_ [10]; however, with its dual phosphatase activity, SynJ1 can potentially act on many different phosphatidylinositol (PI) substrates. Synaptojanin-like proteins in yeast have been found to be involved in multiple trafficking processes such as endocytosis and trans-Golgi sorting [39]–[41]. However, in vertebrate neurons the role of SynJ1 in trafficking events other than clathrin-mediated endocytosis is less clear. In a recent study, the overexpression of SynJ1 in a transgenic mouse line resulted in the accumulation of enlarged early endosomes in neurons [14]. In addition, we have previously shown that SynJ1 protein is present in both synaptic terminals and the ISs of cone photoreceptors, suggesting that it may also have additional functions in PI metabolism at cellular sites other than the synapse [9].

In this study, using TEM imaging, we observed the accumulation of large vesicular structures in the IS of *nrc^a14^* cone photoreceptors at 5 dpf. The vesicular structures in the TEM images are abundant, heterogeneous in size and appearance, and often fill the majority of IS between the nucleus and the mitochondria. In order to investigate the trafficking mechanisms that resulted in this vesicle accumulation, we dark adapted WT and *nrc^a14^* zebrafish larvae. We predicted that increasing synaptic vesicle transport, which is expected to occur in the dark due to increased glutamate release, would enhance appearance of accumulated vesicles. We found that dark adaptation results in an increase in the severity of vesicle accumulation in *nrc^a14^* ISs (Figure 4). As photoreceptors transition between light and dark conditions, they undergo multiple cellular changes including protein translocation [42], [43] and redistribution of energy [44]. The exacerbation of the *nrc^a14^* phenotype in the dark suggests that SynJ1 could play an important role in facilitating changes in vesicle trafficking required for photoreceptor function in the dark. Dark exposure should result in an increase of trafficking of synaptic proteins basally, as well as an increase in the recycling and/or degradation of synaptic vesicle components. Disruption of either of these two trafficking pathways could result in an accumulation of vesicular structures in ISs. To discriminate between these two possibilities, we targeted GFP to subcellular organelles involved in either the biosynthetic or the degradative pathway.

We found that SynJ1 is required for maintaining, but not establishing, the structure of the Golgi apparatus in cone photoreceptors. In the zebrafish larval retina, cone photoreceptors begin to develop around 2 dpf with their OSs and synaptic terminals becoming visible at approximately 2.5 dpf. Synaptic contacts between cone and bipolar cell terminals and the first electroretinogram responses become detectable at 3–3.5 dpf [6], [45]. In the *nrc^a14^* mutant, the Golgi apparatus in the photoreceptors appears to develop normally, but begins to become disordered at approximately 4 dpf; at this time point in development, WT photoreceptor synapses are functional. Release of neurotransmitter at the synaptic terminal requires a constant supply of newly synthesized synaptic proteins from the IS, as well as efficient recycling of these proteins. Dysfunction of either of these trafficking pathways would result in a buildup of synaptic proteins in the IS. The disordered Golgi in *nrc^a14^* photoreceptors would be predicted to cause a defect in trafficking newly synthesized synaptic proteins. Prior to reaching their destinations, many apical and basal proteins both pass through the trans Golgi [46]. However, the ability of *nrc^a14^* cone photoreceptors to maintain proper apical trafficking despite altered Golgi morphology suggests that the disruption of protein transport of newly synthesized proteins is not the primary defect. The developmentally late disruption of the Golgi suggests a secondary defect due to altered endolysosomal trafficking. The Golgi and endolysosomal system exchange proteins and lipids [47] and defects in endolysosomal trafficking caused by genetic diseases [48], [49], or genetic or chemical manipulations [50] can cause alterations in Golgi morphology and function.

All cell types need to maintain protein quality and homeostasis by degrading damaged proteins. However, due to their high levels of membrane turnover and long life, neuronal cell types, including photoreceptors, are severely affected by impairments in endolysosomal trafficking and protein degradation [51]–[53]. Disruptions in endolysosomal and autophagic trafficking have been found in many neurodegenerative diseases including Parkinson’s disease [54]–[57]. In addition, mutations in SynJ1 have recently been linked to a human familial early-onset Parkinsonism [58], [59]. In our current study, we found that *nrc^a14^* cones have multiple abnormalities in the endolysosomal and autophagic systems. These cells contain enlarged acidic vesicles, aberrantly shaped Rab7 positive late endosomes, and an increase in LC3 positive autophagic vesicles (Figure 6). These observations indicate that loss of SynJ1 in cones disrupts late endosomal and autophagic trafficking. In addition, upon dark incubation, LC3-positive puncta appeared larger in *nrc^a14^* mutant cones compared to puncta observed in the light. Thus, changes in both the number and/or shape of vesicles were observed in darkness by both TEM and when using an autophagy marker, which suggests that the vesicles observed in TEM may also be associated with the autophagic trafficking pathway. These findings are consistent with our observation of morphologically normal apical ends of *nrc^a14^* cones; OS disc shedding to remove damaged proteins and lipids does not rely on intracellular endolysosomal pathways. Further, while two synaptic proteins, VAMP2 and RibeyeB, mislocalize to the ISs of *nrc^a14^* photoreceptors, a third synaptic protein, Synaptophysin, does not. Synaptophysin protein can be polyubiquitinated and degraded by the proteasome [60], and therefore this protein may be able to bypass defects in the endolysosomal system.

Regulation of endosomal and autophagic trafficking is highly regulated by phosphoinositides, and disrupting the activity of phosphoinositide kinases and phosphatases can result in aberrant endosomal trafficking and enlarged endosomal structures [31], [61], [62]. The phosphoinositide PI(3,5)P_2_ is enriched on late endosome/lysosome membranes. Mice with genetic mutations affecting the phosphatases and kinases involved in regulation of PI(3,5)P_2_ have neurological defects, cells with large vacuoles, abnormal endolysosomal membranes [63]–[66], and defective autophagy [67]. Similarly, PI3P is enriched on autophagosome membranes and deletion or mutation of PI3P modulating enzymes result in aberrant autophagy [68]. The yeast synaptojanin-like proteins INP52 and INP53 can dephosphorylate both PI(3,5)P_2_ and PI3P to PI [69]. The phosphatase domains of SynJ1 are highly conserved from yeast to vertebrates; while the role of SynJ1 in regulating these phosphoinositide species has not been investigated *in vivo*, mouse and human SynJ1 constructs can act on PI(3)P *in vitro* [58], [69], [70]. Further work will need to be done to characterise the PI species affected *in vivo* by the *nrc^a14^* mutation.

## Materials and Methods

### Ethics Statement

This study was carried out in strict accordance with the recommendations in the Guide for the Care and Use of Laboratory Animals of the National Institutes of Health. The protocol, 3113-01, was approved by IACUC of the University of Washington.

### Cloning and Plasmids

The medial Golgi marker, *TαCP:zfMan2a-GFP*, was obtained from Brian Link [71]. The synaptic vesicle marker, *CFP-zfSynaptophysin*, was obtained from Martin Meyer [25]. The autophagosome marker, *GFP-zfLC3* was obtained from Dan Klionsky [32] and cloned into a pCR8/GW Gateway vector (Invitrogen). The ER marker ER-GFP was created by overhang PCR methods to add a C-terminal KDEL sequence, and N-terminal 17 amino acids (MTALSLLFMAVSVALIT) of zfcalreticulin to GFP (based on [28]) and cloned into a pCR8/GW Gateway vector (Invitrogen). *zfRab7* was cloned from larval zebrafish cDNA using the primers 5′-ATTCGCTAGATCTCCTGCTTT-3′ and 5′-AGGCTGAGGGTGAAATGTTG-3′, and cloned into a 3′Entry Vector (Invitrogen) using the primers 5′-GGGGACAGCTTTCTTGTACAAAGTGGCTATGACATCAAGGAAGAAAGT-3′ and 5′-GGGGACAACTTTGTATAATAAAGTTGCTCAGCAGCTACAGGTCTCTG-3′. Expression constructs were generated using the MultiSite Gateway System (Invitrogen) and the Tol2 kit [72]. Expression was driven by either the *cone transducin alpha* promoter (*TαCP*) [22] or the *cone-rod homeobox* promoter (*crx*). To obtain the *crx* promoter, a 5 kb upstream fragment of *crx* was amplified from zebrafish genomic DNA by PCR using the following primers, 5′-GGGGACAACTTTGTATAGAAAAGTTGTCATGGAAATGGCAAAAACATTC-3′ and 5′-GGGGACTGCTTTTTTGTACAAACTTGGCGCTACTCGCTGTCTTCAATAA-3′. The resultant PCR DNA fragment was subcloned into pDONR P4P1r by Gateway BP reaction [23].

### Fish Husbandry and Generation of Transgenic Zebrafish

Zebrafish were reared and maintained in the University of Washington fish facility as previously described [73]. Embryos were maintained in embryo media (EM) [73] at 28°C on a 14/10 hour light/dark cycle prior to experimentation or rearing in the fish facility. Homozygous *nrc^a14^* mutants were identified by the OKR as previously described [74] or by genotyping. Genotyping was performed by amplifying the region of the *synJ1* gene containing the *nrc^a14^* mutation site form genomic DNA using the primers 5′-CACCAGAACCATCCAGAACA-3′ and 5′-GTCATACCGCTCAGCCCTAA-3′. The *nrc^a14^* mutation disrupts a cut site recognition sequence of the restriction enzyme BssSI, allowing *nrc^a14^* mutants to be identified. Since WT and *nrc^a14^* heterozygotes appear indistinguishable in every phenotypic assay we have performed, we refer to all OKR-positive larvae as WT. The *Tg(TαCP:*spH) fish line has been previously described [9]. We created other transgenic fish lines by injecting DNA expression constructs described above and mRNA encoding the tol2 transposase simultaneously into zebrafish embryos at the one-cell stage. Once injected zebrafish reached sexual maturity, pair-wise crosses were performed to identify fish that could transmit the desired transgene [75]. Newly identified transgenic fish were assayed by OKR at 5 dpf to ensure that transgene expression did not affect visual responses. For dark adaptation, larvae were raised in the dark for 24 hrs starting at 4 dpf. Light-adapted WT and *nrc^a14^* larvae were kept on a normal 14/10 hour light/dark cycle. The 24 hr incubations started in the afternoon. After 24 hrs (at 5 dpf) dark-adapted larvae were fixed in the dark, light-adapted controls were fixed in the light, and then fixed larvae were processed for TEM or histology.

### Immunohistochemistry

Retinal slices were prepared as previously described [76]. After blocking in 5% normal goat serum, 1% bovine serum albumin, and 0.3% TritonX-100 in PBS, slices were incubated with the following primary antibodies at 4°C overnight: 1∶100 anti-RibeyeB (a gift from Teresa Nicolson, [77]), 1∶5000 anti-IFT88 (a gift from Brian Perkins, [21]), or 1∶500 anti-acetylated tubulin (Sigma, T6793). Slices were incubated with secondary anti-rabbit Alexa-633 (Invitrogen, A21071) or anti-mouse Alexa 568 (Invitrogen, A11002) at 1∶200 for 1 hour at room temperature. Nuclei were counter stained with 5 µM Sytox Green (Invitrogen) or 5 µM Hoechst (Invitrogen). Slides were mounted with a coverslip and Fluoromount-G (Southern Biotech). Imaging of retinal sections was performed on an Olympus FV300 or FV1000 confocal microscope with a 60X oil immersion objective.

### Live Imaging

Larvae were treated with 0.003% 1-phenyl-2-thiourea (PTU) in EM at ∼24 hours post fertilization (hpf) to prevent melanization [73]. For membrane counterstaining, 3 dpf larvae were incubated in 25 µM BODIPY-Texas Red (Invitrogen) for 1 hour, followed by three washes in EM with PTU prior to imaging. For Brefeldin A treatments, 3 dpf larvae were incubated with 2 µM Brefeldin A (Cell Signalling) in EM and PTU for 90 minutes prior to imaging. For Lysotracker Red staining, 5 dpf larvae were incubated in 10 µM Lysotracker Red DND 99 (Invitrogen) in EM and PTU for 2 hours, followed by three washes in EM and PTU prior to imaging. Larvae were anaesthetized in Tricaine (Sigma) and mounted in warm 0.5–1% low mount agarose. Embedded larvae were covered in EM containing PTU and Tricaine and imaged. For BFA treatments, 2 µM BFA was included in the EM during imaging. Imaging was done on an Olympus FV1000 using a 40X or 60X water immersion objective.

### Transmission Electron Microscopy

Transmission electron microscopy was performed at the UW Vision Core as previously described [45]. For quantification, the entire retina was imaged.

### Image Processing and Data Analysis

Images were processed using NIH ImageJ and Adobe Photoshop. Representative images in Figures are 2 µm projections of confocal stacks, or single optical slices of TEM images. Images were randomized prior to analysis. ImageJ was used to measure CC and OS lengths and vesicles sizes from randomized images. For qualitative comparisons between WT and *nrc* ^a14^ larvae, at least 6 larvae of each genotype were analyzed. For quantitative data, the number of larvae analyzed is included in the text and Figure legends. For light/dark experiments cells were scored as “vesiculated” if they contained at least three large (>125 nm in diameter) vesicular structures in their ISs. If the number of vesicular structures exceeded 20, the cell was scored as “severely vesiculated”. Microsoft Excel and R were used for statistical analysis. Unless otherwise indicated, bar graphs and values in text represent mean values +/− STDEV and p-values were calculated by the Mann-Whitney test.

## Supporting Information

Figure S1 **RibeyeB is mislocalized in** ***nrc^a14^*** **inner segments even in the absence of** ***TαCP:spH*** **.** Anti-RibeyeB staining of non-transgenic WT and *nrc^a14^* 5 dpf retinas showed the same staining pattern as *Tg(TαCP:spH)* 5 dpf retinas in Figure 2. In WT photoreceptors, RibeyeB was found only at synaptic terminals (A). In *nrc^a14^* cone photoreceptors, the RibeyeB staining was visible in both the synaptic terminals and ISs (B). Anti-RibeyeB staining is shown in magenta and Hoechst stained nuclei are in blue. Syn = photoreceptor synapses, IS = inner segment. Scale bar = 2 µm in all images.
(TIF)

Figure S2 **Man2a-GFP marks medial Golgi structures.** We confirmed that our Golgi marker behaved in the same manner as endogenous Golgi proteins by treating 3 dpf *Tg(crx:Man2a-GFP)* zebrafish larvae with 2 µM Brefeldin A followed by live imaging. After a 4 hour incubation in BFA, the GFP signal was present in the ER and fragmented Golgi structures.
(TIF)

## References

[1] Kolb H (2003) How the retina works - Much of the construction of an image takes place in the retina itself through the use of specialized neural circuits. American Scientist 91: 28–35.

[2] Kennedy B, Malicki J (2009) What drives cell morphogenesis: a look inside the vertebrate photoreceptor. Dev Dyn 238: 2115–2138. 19582864 10.1002/dvdy.22010 PMC2772090

[3] Young RW (1967) The renewal of photoreceptor cell outer segments. J Cell Biol 33: 61–72. 6033942 10.1083/jcb.33.1.61 PMC2107286

[4] Ramamurthy V, Cayouette M (2009) Development and disease of the photoreceptor cilium. Clin Genet 76: 137–145. 19790290 10.1111/j.1399-0004.2009.01240.x

[5] Sung CH, Chuang JZ (2010) The cell biology of vision. J Cell Biol 190: 953–963. 20855501 10.1083/jcb.201006020 PMC3101587

[6] Allwardt BA, Lall AB, Brockerhoff SE, Dowling JE (2001) Synapse formation is arrested in retinal photoreceptors of the zebrafish *nrc* mutant. J Neurosci 21: 2330–2342. 11264308 10.1523/JNEUROSCI.21-07-02330.2001 PMC6762396

[7] Van Epps HA, Yim CM, Hurley JB, Brockerhoff SE (2001) Investigations of photoreceptor synaptic transmission and light adaptation in the zebrafish visual mutant *nrc* . Invest Ophthalmol Vis Sci 42: 868–874. 11222552

[8] Van Epps HA, Hayashi M, Lucast L, Stearns GW, Hurley JB, et al (2004) The zebrafish *nrc* mutant reveals a role for the polyphosphoinositide phosphatase synaptojanin 1 in cone photoreceptor ribbon anchoring. J Neurosci 24: 8641–8650. 15470129 10.1523/JNEUROSCI.2892-04.2004 PMC6729946

[9] Holzhausen LC, Lewis AA, Cheong KK, Brockerhoff SE (2009) Differential role for synaptojanin 1 in rod and cone photoreceptors. J Comp Neurol 517: 633–644. 19827152 10.1002/cne.22176 PMC3071606

[10] Cremona O, Di Paolo G, Wenk MR, Luthi A, Kim WT, et al (1999) Essential role of phosphoinositide metabolism in synaptic vesicle recycling. Cell 99: 179–188. 10535736 10.1016/s0092-8674(00)81649-9

[11] McPherson PS, Garcia EP, Slepnev VI, David C, Zhang X, et al (1996) A presynaptic inositol-5-phosphatase. Nature 379: 353–357. 8552192 10.1038/379353a0

[12] McPherson PS, Takei K, Schmid SL, De Camilli P (1994) p145, a major Grb2-binding protein in brain, is co-localized with dynamin in nerve terminals where it undergoes activity-dependent dephosphorylation. J Biol Chem 269: 30132–30139. 7982917

[13] Gong LW, De Camilli P (2008) Regulation of postsynaptic AMPA responses by synaptojanin 1. Proc Natl Acad Sci U S A 105: 17561–17566. 18987319 10.1073/pnas.0809221105 PMC2579885

[14] Cossec JC, Lavaur J, Berman DE, Rivals I, Hoischen A, et al (2012) Trisomy for synaptojanin1 in Down syndrome is functionally linked to the enlargement of early endosomes. Hum Mol Genet 21: 3156–3172. 22511594 10.1093/hmg/dds142 PMC3384382

[15] Wright AF, Chakarova CF, El-Aziz MMA, Bhattacharya SS (2010) Photoreceptor degeneration: genetic and mechanistic dissection of a complex trait. Nature Reviews Genetics 11: 273–284. 10.1038/nrg2717 20212494

[16] Insinna C, Besharse JC (2008) Intraflagellar transport and the sensory outer segment of vertebrate photoreceptors. Dev Dyn 237: 1982–1992. 18489002 10.1002/dvdy.21554 PMC2692564

[17] Branchek T (1984) The development of photoreceptors in the zebrafish, *brachydanio rerio*. II. Function. J Comp Neurol 224: 116–122. 6715575 10.1002/cne.902240110

[18] Easter SS Jr, Nicola GN (1996) The development of vision in the zebrafish (*Danio rerio*). Dev Biol 180: 646–663. 8954734 10.1006/dbio.1996.0335

[19] Brockerhoff SE, Hurley JB, Janssen-Bienhold U, Neuhauss SC, Driever W, et al (1995) A behavioral screen for isolating zebrafish mutants with visual system defects. Proc Natl Acad Sci U S A 92: 10545–10549. 7479837 10.1073/pnas.92.23.10545 PMC40648

[20] Tsujikawa M, Malicki J (2004) Intraflagellar transport genes are essential for differentiation and survival of vertebrate sensory neurons. Neuron 42: 703–716. 15182712 10.1016/s0896-6273(04)00268-5

[21] Krock BL, Perkins BD (2008) The intraflagellar transport protein IFT57 is required for cilia maintenance and regulates IFT-particle-kinesin-II dissociation in vertebrate photoreceptors. J Cell Sci 121: 1907–1915. 18492793 10.1242/jcs.029397 PMC2637114

[22] Kennedy BN, Alvarez Y, Brockerhoff SE, Stearns GW, Sapetto-Rebow B, et al (2007) Identification of a zebrafish cone photoreceptor-specific promoter and genetic rescue of achromatopsia in the *nof* mutant. Invest Ophthalmol Vis Sci 48: 522–529. 17251445 10.1167/iovs.06-0975

[23] Miesenbock G, De Angelis DA, Rothman JE (1998) Visualizing secretion and synaptic transmission with pH-sensitive green fluorescent proteins. Nature 394: 192–195. 9671304 10.1038/28190

[24] Wan L, Almers W, Chen W (2005) Two ribeye genes in teleosts: the role of Ribeye in ribbon formation and bipolar cell development. J Neurosci 25: 941–949. 15673675 10.1523/JNEUROSCI.4657-04.2005 PMC6725632

[25] Meyer MP, Smith SJ (2006) Evidence from in vivo imaging that synaptogenesis guides the growth and branching of axonal arbors by two distinct mechanisms. J Neurosci 26: 3604–3614. 16571769 10.1523/JNEUROSCI.0223-06.2006 PMC6673851

[26] Mazelova J, Ransom N, Astuto-Gribble L, Wilson MC, Deretic D (2009) Syntaxin 3 and SNAP-25 pairing, regulated by omega-3 docosahexaenoic acid, controls the delivery of rhodopsin for the biogenesis of cilia-derived sensory organelles, the rod outer segments. J Cell Sci 122: 2003–2013. 19454479 10.1242/jcs.039982 PMC2723154

[27] Trapani JG, Obholzer N, Mo W, Brockerhoff SE, Nicolson T (2009) Synaptojanin1 is required for temporal fidelity of synaptic transmission in hair cells. PLoS Genet 5: e1000480. 19424431 10.1371/journal.pgen.1000480 PMC2673039

[28] Roderick HL, Campbell AK, Llewellyn DH (1997) Nuclear localisation of calreticulin in vivo is enhanced by its interaction with glucocorticoid receptors. FEBS Lett 405: 181–185. 9089287 10.1016/s0014-5793(97)00183-x

[29] Suzuki SC, Bleckert A, Williams PR, Takechi M, Kawamura S, et al (2013) Cone photoreceptor types in zebrafish are generated by symmetric terminal divisions of dedicated precursors. Proc Natl Acad Sci U S A 110: 15109–14. 23980162 10.1073/pnas.1303551110 PMC3773785

[30] Deretic D, Papermaster DS (1991) Polarized sorting of rhodopsin on post-Golgi membranes in frog retinal photoreceptor cells. J Cell Biol 113: 1281–1293. 1828467 10.1083/jcb.113.6.1281 PMC2289036

[31] Vicinanza M, D'Angelo G, Di Campli A, De Matteis MA (2008) Function and dysfunction of the PI system in membrane trafficking. EMBO J 27: 2457–2470. 18784754 10.1038/emboj.2008.169 PMC2536629

[32] He C, Bartholomew CR, Zhou W, Klionsky DJ (2009) Assaying autophagic activity in transgenic GFP-Lc3 and GFP-Gabarap zebrafish embryos. Autophagy 5: 520–526. 19221467 10.4161/auto.5.4.7768 PMC2754832

[33] Vitelli R, Santillo M, Lattero D, Chiariello M, Bifulco M, et al (1997) Role of the small GTPase Rab7 in the late endocytic pathway. J Biol Chem 272: 4391–4397. 9020161 10.1074/jbc.272.7.4391

[34] Bucci C, Thomsen P, Nicoziani P, McCarthy J, van Deurs B (2000) Rab7: a key to lysosome biogenesis. Mol Biol Cell 11: 467–480. 10679007 10.1091/mbc.11.2.467 PMC14786

[35] Jager S, Bucci C, Tanida I, Ueno T, Kominami E, et al (2004) Role for Rab7 in maturation of late autophagic vacuoles. J Cell Sci 117: 4837–4848. 15340014 10.1242/jcs.01370

[36] Bottger G, Nagelkerken B, van der Sluijs P (1996) Rab4 and Rab7 define distinct nonoverlapping endosomal compartments. J Biol Chem 271: 29191–29197. 8910576 10.1074/jbc.271.46.29191

[37] Klionsky DJ, Abdalla FC, Abeliovich H, Abraham RT, Acevedo-Arozena A, et al (2012) Guidelines for the use and interpretation of assays for monitoring autophagy. Autophagy 8: 445–544. 22966490 10.4161/auto.19496 PMC3404883

[38] Hartong DT, Berson EL, Dryja TP (2006) Retinitis pigmentosa. Lancet 368: 1795–1809. 17113430 10.1016/S0140-6736(06)69740-7

[39] Stefan CJ, Audhya A, Emr SD (2002) The yeast synaptojanin-like proteins control the cellular distribution of phosphatidylinositol (4,5)-bisphosphate. Mol Biol Cell 13: 542–557. 11854411 10.1091/mbc.01-10-0476 PMC65648

[40] Bensen ES, Costaguta G, Payne GS (2000) Synthetic genetic interactions with temperature-sensitive clathrin in *Saccharomyces cerevisiae*. Roles for synaptojanin-like Inp53p and dynamin-related Vps1p in clathrin-dependent protein sorting at the trans-Golgi network. Genetics 154: 83–97. 10628971 10.1093/genetics/154.1.83 PMC1460916

[41] Singer-Kruger B, Nemoto Y, Daniell L, Ferro-Novick S, De Camilli P (1998) Synaptojanin family members are implicated in endocytic membrane traffic in yeast. J Cell Sci 111 3347–3356. 9788876 10.1242/jcs.111.22.3347

[42] Arshavsky VY (2003) Protein Translocation in Photoreceptor Light Adaptation: A Common Theme in Vertebrate and Invertebrate Vision. Sci STKE 204: pe43. 10.1126/stke.2003.204.pe43 14560045

[43] Rajala A, Daly RJ, Tanito M, Allen DT, Holt LJ, et al (2009) Growth factor receptor-bound protein 14 undergoes light-dependent intracellular translocation in rod photoreceptors: functional role in retinal insulin receptor activation. Biochemistry 48: 5563–5572. 19438210 10.1021/bi9000062 PMC2763493

[44] Linton JD, Holzhausen LC, Babai N, Song H, Miyagishima KJ, et al (2010) Flow of energy in the outer retina in darkness and in light. Proc Natl Acad Sci U S A 107: 8599–8604. 20445106 10.1073/pnas.1002471107 PMC2889335

[45] Schmitt EA, Dowling JE (1999) Early retinal development in the zebrafish, *Danio rerio*: light and electron microscopic analyses. J Comp Neurol 404: 515–536. 9987995

[46] Schmied R, Holtzman E (1989) Involvement of the Golgi apparatus in sorting of materials to opposite ends of frog rod retinal photoreceptors. Journal of Neurobiology 20: 115–138. 2785160 10.1002/neu.480200303

[47] Pfeffer SR (2009) Multiple routes of protein transport from endosomes to the trans Golgi network. FEBS Lett 583: 3811–3816. 19879268 10.1016/j.febslet.2009.10.075 PMC2787657

[48] Ganley IG, Pfeffer SR (2006) Cholesterol accumulation sequesters Rab9 and disrupts late endosome function in NPC1-deficient cells. J Biol Chem 281: 17890–17899. 16644737 10.1074/jbc.M601679200 PMC3650718

[49] Choudhury A, Dominguez M, Puri V, Sharma DK, Narita K, et al (2002) Rab proteins mediate Golgi transport of caveola-internalized glycosphingolipids and correct lipid trafficking in Niemann-Pick C cells. J Clin Invest 109: 1541–1550. 12070301 10.1172/JCI15420 PMC151017

[50] Kirkbride KC, Hong NH, French CL, Clark ES, Jerome WG, et al (2012) Regulation of late endosomal/lysosomal maturation and trafficking by cortactin. Cytoskeleton (Hoboken) 69: 625–643. 22991200 10.1002/cm.21051 PMC3746372

[51] Dermaut B, Norga KK, Kania A, Verstreken P, Pan H, et al (2005) Aberrant lysosomal carbohydrate storage accompanies endocytic defects and neurodegeneration in Drosophila benchwarmer. J Cell Biol 170: 127–139. 15998804 10.1083/jcb.200412001 PMC2171373

[52] Chinchore Y, Mitra A, Dolph PJ (2009) Accumulation of rhodopsin in late endosomes triggers photoreceptor cell degeneration. PLoS Genet 5: e1000377. 19214218 10.1371/journal.pgen.1000377 PMC2633617

[53] Nixon RA, Yang DS, Lee JH (2008) Neurodegenerative lysosomal disorders: a continuum from development to late age. Autophagy 4: 590–599. 18497567 10.4161/auto.6259

[54] Dehay B, Martinez-Vicente M, Caldwell GA, Caldwell KA, Yue Z, et al (2013) Lysosomal impairment in Parkinson's disease. Mov Disord 28: 725–732. 23580333 10.1002/mds.25462 PMC5131721

[55] Tofaris GK (2012) Lysosome-dependent pathways as a unifying theme in Parkinson's disease. Mov Disord 27: 1364–1369. 22927213 10.1002/mds.25136

[56] Nixon RA (2013) The role of autophagy in neurodegenerative disease. Nat Med 19: 983–997. 23921753 10.1038/nm.3232

[57] Cook C, Stetler C, Petrucelli L (2012) Disruption of protein quality control in Parkinson's disease. Cold Spring Harb Perspect Med 2: a009423. 22553500 10.1101/cshperspect.a009423 PMC3331692

[58] Krebs CE, Karkheiran S, Powell JC, Cao M, Makarov V, et al (2013) The Sac1 Domain of SYNJ1 Identified Mutated in a Family with Early-Onset. Hum Mutat 34: 1200–1207. 23804563 10.1002/humu.22372 PMC3790461

[59] Quadri M, Fang M, Picillo M, Olgiati S, Breedveld GJ, et al (2013) Mutation in the *SYNJ1* Gene Associated with Autosomal Recessive, Early-Onset. Hum Mutat 34: 1208–1215. 23804577 10.1002/humu.22373

[60] Wheeler TC, Chin L-S, Li Y, Roudabush FL, Li L (2002) Regulation of Synaptophysin Degradation by Mammalian Homologues of Seven in Absentia. J Biol Chem 277: 10273–10282. 11786535 10.1074/jbc.M107857200

[61] Vicinanza M, D'Angelo G, Di Campli A, De Matteis MA (2008) Phosphoinositides as regulators of membrane trafficking in health and disease. Cell Mol Life Sci 65: 2833–2841. 18726176 10.1007/s00018-008-8353-2 PMC11131623

[62] Ooms LM, Horan KA, Rahman P, Seaton G, Gurung R, et al (2009) The role of the inositol polyphosphate 5-phosphatases in cellular function and human disease. Biochem J 419: 29–49. 19272022 10.1042/BJ20081673

[63] Ikonomov OC, Sbrissa D, Delvecchio K, Xie Y, Jin JP, et al (2011) The phosphoinositide kinase PIKfyve is vital in early embryonic development: Preimplantation lethality of *PIKfyve−/−* embryos but normality of *PIKfyve+/−* mice. J Biol Chem 286: 13404–13413. 21349843 10.1074/jbc.M111.222364 PMC3075686

[64] Chow CY, Zhang Y, Dowling JJ, Jin N, Adamska M, et al (2007) Mutation of FIG4 causes neurodegeneration in the pale tremor mouse and patients with CMT4J. Nature 448: 68–72. 17572665 10.1038/nature05876 PMC2271033

[65] Ferguson CJ, Lenk GM, Jones JM, Grant AE, Winters JJ, et al (2012) Neuronal expression of Fig4 is necessary and sufficient to prevent spongiform neurodegeneration. Hum Mol Genet 21: 3525–3534. 22581779 10.1093/hmg/dds179 PMC3406753

[66] Zhang Y, Zolov SN, Chow CY, Slutsky SG, Richardson SC, et al (2007) Loss of Vac14, a regulator of the signalling lipid phosphatidylinositol 3,5-bisphosphate, results in neurodegeneration in mice. Proc Natl Acad Sci U S A 104: 17518–17523. 17956977 10.1073/pnas.0702275104 PMC2077288

[67] Ferguson CJ, Lenk GM, Meisler MH (2009) Defective autophagy in neurons and astrocytes from mice deficient in PI(3,5)P2. Hum Mol Genet 18: 4868–4878. 19793721 10.1093/hmg/ddp460 PMC2778378

[68] Vergne I, Deretic V (2010) The role of PI3P phosphatases in the regulation of autophagy. FEBS Lett 584: 1313–1318. 20188094 10.1016/j.febslet.2010.02.054 PMC2885894

[69] Guo S, Stolz LE, Lemrow SM, York JD (1999) SAC1-like domains of yeast SAC1, INP52, and INP53 and of human synaptojanin encode polyphosphoinositide phosphatases. J Biol Chem 274: 12990–12995. 10224048 10.1074/jbc.274.19.12990

[70] Mani M, Lee SY, Lucast L, Cremona O, Di Paolo G, et al (2007) The dual phosphatase activity of synaptojanin1 is required for both efficient synaptic vesicle endocytosis and reavailability at nerve terminals. Neuron 56: 1004–1018. 18093523 10.1016/j.neuron.2007.10.032 PMC3653591

[71] Insinna C, Baye LM, Amsterdam A, Besharse JC, Link BA (2010) Analysis of a zebrafish *dync1h1* mutant reveals multiple functions for cytoplasmic dynein 1 during retinal photoreceptor development. Neural Dev 5: 1–21. 20412557 10.1186/1749-8104-5-12 PMC2880287

[72] Kwan KM, Fujimoto E, Grabher C, Mangum BD, Hardy ME, et al (2007) The Tol2kit: a multisite gateway-based construction kit for Tol2 transposon transgenesis constructs. Dev Dyn 236: 3088–3099. 17937395 10.1002/dvdy.21343

[73] Westerfield M (1995) The Zebrafish Book: A Guide for the Laboratory Use of Zebrafish (*Brachydanio rerio)*. Eugene: University of Oregon Press.

[74] Brockerhoff SE (2006) Measuring the optokinetic response of zebrafish larvae. Nat Protoc 1: 2448–2451. 17406490 10.1038/nprot.2006.255

[75] Kikuta H, Kawakami K (2009) Transient and stable transgenesis using tol2 transposon vectors. Methods Mol Biol 546: 69–84. 19378098 10.1007/978-1-60327-977-2_5

[76] Brockerhoff SE, Hurley JB, Niemi GA, Dowling JE (1997) A new form of inherited red-blindness identified in zebrafish. J Neurosci 20: 1–8. 10.1523/JNEUROSCI.17-11-04236.1997 PMC6573554 9151740

[77] Obholzer N, Wolfson S, Trapani JG, Mo W, Nechiporuk A, et al (2008) Vesicular glutamate transporter 3 is required for synaptic transmission in zebrafish hair cells. J Neurosci 28: 2110–2118. 18305245 10.1523/JNEUROSCI.5230-07.2008 PMC6671858

