# Supplementary figures and images for: Synaptojanin 1 Is Required for Endolysosomal Trafficking of Synaptic Proteins in Cone Photoreceptor Inner Segments
Source: PLoS One. 2014 Jan 2;9(1):e84394. doi: 10.1371/journal.pone.0084394 (PMC3879297; doi:10.1371/journal.pone.0084394)

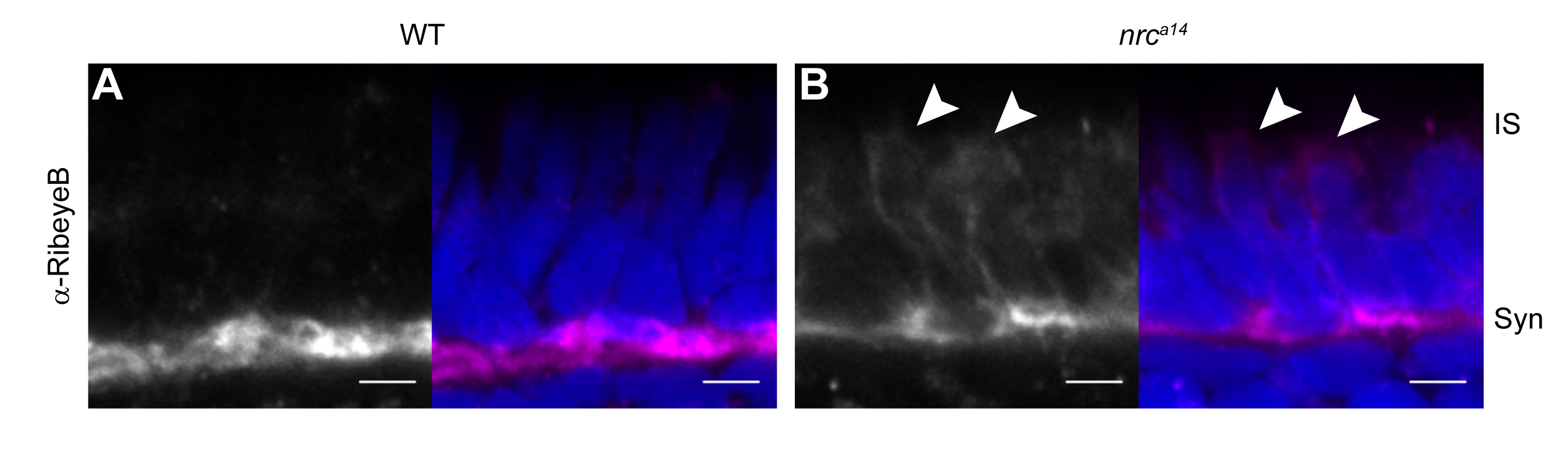

Supplement: Figure S1 — RibeyeB is mislocalized in nrca14 inner segments even in the absence of TαCP:spH . Anti-RibeyeB staining of non-transgenic WT and nrca14 5 dpf retinas showed the same staining pattern as Tg(TαCP:spH) 5 dpf retinas in Figure 2. In WT photoreceptors, RibeyeB was found only at synaptic terminals (A). In nrca14 cone photoreceptors, the RibeyeB staining was visible in both the synaptic terminals and ISs (B). Anti-RibeyeB staining is shown in magenta and Hoechst stained nuclei are in blue. Syn = photoreceptor synapses, IS = inner segment. Scale bar = 2 µm in all images. (TIF) [file pone.0084394.s001.tif]

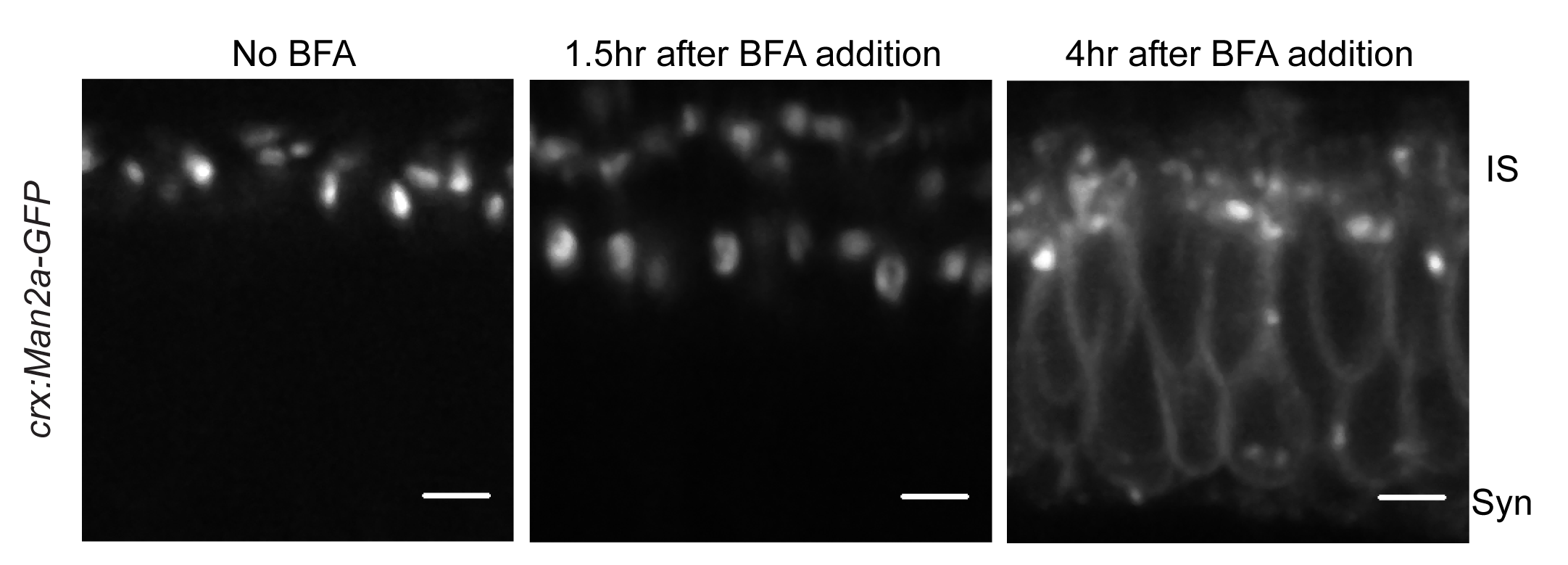

Supplement: Figure S2 — Man2a-GFP marks medial Golgi structures. We confirmed that our Golgi marker behaved in the same manner as endogenous Golgi proteins by treating 3 dpf Tg(crx:Man2a-GFP) zebrafish larvae with 2 µM Brefeldin A followed by live imaging. After a 4 hour incubation in BFA, the GFP signal was present in the ER and fragmented Golgi structures. (TIF) [file pone.0084394.s002.tif]
